# Incidence of Sickle Cell Trait — United States, 2010

**Published:** 2014-12-12

**Authors:** Jelili Ojodu, Mary M. Hulihan, Shammara N. Pope, Althea M. Grant

**Affiliations:** 1 Association of Public Health Laboratories; 2 Division of Blood Disorders, National Center for Birth Defects and Developmental Disabilities, CDC

Persons with sickle cell trait (SCT) are heterozygous carriers of an abnormal ß-globin gene that results in the production of an abnormal hemoglobin, Hb S, which can distort red blood cells (http://www.cdc.gov/ncbddd/sicklecell/facts.html). All state newborn screening (NBS) programs have provided universal sickle cell disease (SCD) screening for newborns since 2006. Screening for SCD detects both SCD and SCT. To obtain up-to-date measures of the occurrence of SCT among newborns by race/ethnicity and state of birth, data collected by state NBS programs in 2010 were examined. In 2010, the incidence of SCT in participating states was 15.5 per 1,000 newborns overall; 73.1 among black newborns and 6.9 among Hispanic newborns. Incidence by state ranged from 0.8 per 1,000 screened newborns in Montana to 34.1 per 1,000 in Mississippi. Although the occurrence of SCT varies greatly from state-to-state and among different races and ethnicities, every state and racial/ethnic population includes persons living with the condition. The period immediately following NBS is ideal for primary care providers and genetic counselors to begin educating the families of identified persons with SCT about potential health complications and reproductive considerations.

State NBS programs were requested via e-mail by CDC investigators to provide aggregate data on the total number of infants screened in 2010 and the total number with a positive SCT result. Data were also requested to allow categorizing the births by Hispanic ethnicity* and by race.† At least four attempts were made to obtain the data (three e-mails and one telephone call). A total of 44 states provided data, of which 17 also provided ethnicity and/or race information: 13 states provided ethnicity categories for >90% of the infants, and 13 states provided race categories for >90% of the infants. The incidence of SCT was calculated for each state, overall, and by ethnicity and race, when possible. States did not provide data for combined racial/ethnic categories; that is, Hispanic ethnicity includes all races (e.g., black and white), so that a newborn Hispanic infant with a positive SCT result would be included in the calculations for both Hispanics and its race.

In the 44 states for which data were available, there were 55,258 infants with a positive SCT screening result in 2010 (Table 1), or 1.5% of all infants screened. These states represent approximately 88% of the U.S. population, so it is likely that the total number of incident cases for that year in the United States exceeded 60,000. Montana had the lowest incidence of SCT (0.8 cases per 1,000 screened), and Mississippi had the highest incidence (34.1 cases per 1,000 screened). The overall incidence in the population of the 44 states that provided data was 15.5 cases per 1,000 screened. Idaho, Montana, New Hampshire, North Dakota, and Vermont each had fewer than 50 infants with a positive SCT test result, whereas Florida and New York each had more than 5,000.

A total of 17 states also provided SCT results categorized by ethnicity only, race only, or both race and ethnicity. The overall incidence for the 13 states that provided ethnicity data was 6.9 cases per 1,000 Hispanic infants screened (Table 2). The overall incidence for the 13 states that provided race data was 2.2 cases per 1,000 Asian, Native Hawaiian, or other Pacific Islander infants screened; 73.1 cases per 1,000 black or African American infants screened; and 3.0 cases per 1,000 white infants screened (Table 3).

## Discussion

In 1987, the National Institutes of Health convened a consensus development conference on Newborn Screening for Sickle Cell Disease and Other Hemoglobinopathies. The conference attendees, experts in hemoglobinopathies, recommended universal screening for hemoglobinopathies for all U.S. newborns. They also recommended that families of children identified with SCT during the NBS process should receive information to help them understand the differences between carrying one gene (SCT) and carrying two genes (SCD), and that there might be implications for family planning by the parents, and eventually by the newborn (http://consensus.nih.gov/1987/1987ScreeningSickleHemoglobinopathies061html.htm).

There are no standardized methods for reporting positive SCT results to doctors or families of affected persons. A 2007 study found that newborn screening programs provided SCT results to the newborn’s primary care provider in 88% of states, to the birth hospital in 63% of states, to the family in 37% of states, and the results were not reported at all in 4% of states. For programs that reported the positive SCT results, 37% had no mechanism to determine whether or not that information was received by the intended recipient (1). This suggests that opportunities to educate families about the potential health effects of SCT and the implications for future reproductive decisions might have been missed. In addition, there might be consequences for the infant’s own family planning, and it might also have an impact on other children of those parents or their extended family members (2). Each person with SCT identified by screening represents an opportunity to educate a family about the possible health outcomes associated with SCT and the potential for having another child with SCT or SCD. A previous study showed that such families welcomed genetic counseling and health education (3).

What is already known on this topic?

The *National Newborn Screening 10-Year Incidence Report* provided an estimated incidence of sickle cell trait, nationally and by state, for the years 1991–2000. The overall U.S. incidence estimate for sickle cell trait was 15.5 cases per 1,000 births.

What is added by this report?

In 2010, the total U.S. incidence estimate was 15.5 cases per 1,000 births, ranging from 0.8 cases per 1,000 births in Montana to 34.1 cases per 1,000 births in Mississippi. The total U.S. incidence estimate by race only (based on information provided by 13 states) was 73.1 cases per 1,000 black births, 3.0 cases per 1,000 white births, 2.2 cases per 1,000 Asian or Native Hawaiian or Other Pacific Islander births, and by ethnicity only (13 states) was 6.9 cases per 1,000 Hispanic births.

What are the implications for public health practice?

The incidence of sickle cell trait greatly varies from state-to-state and among different races and ethnicities; however, every state and racial/ethnic population has persons living with the condition. The period immediately after newborn screening is ideal for primary care providers and genetic counselors to begin educating the families of identified persons with sickle cell trait about potential health complications and reproductive considerations.

The *National Newborn Screening 10-Year Incidence Report* provided an estimated incidence of SCT, nationally, and by state, for the years 1991–2000 (http://genes-r-us.uthscsa.edu/newborn_reports). In that report, the estimate of SCT incidence ranged from 0.3 cases per 1,000 births in Kentucky to 48.2 cases per 1,000 births in the District of Columbia; the total U.S. incidence estimate was 15.5 cases per 1,000 births (based on data from 45 states and the District of Columbia). As of May 1, 2006, all 50 states and the District of Columbia had implemented universal newborn screening for sickle cell disease and, consequently, SCT (4). This *MMWR* report updates the data that were previously available in the National Newborn Screening Report and estimates that over 60,000 infants were born with SCT in 2010.

Previous studies using data from a single state (5) or from a few counties (6) estimated that SCT was present in approximately 7% of blacks or African Americans. These NBS results show that the incidence ranged from 4.0% of black births in Montana to 10.1% in Michigan and was 7.3% overall in the 13 participating states. Also in comparison with single-state statistics showing an incidence of 0.2% in white infants and 0.5% in Hispanic newborns (5), these results ranged from zero to 0.4% in whites and 0.2% to 6.3% in Hispanics. These NBS results underscore the differences between states that reflect the ancestry of their inhabitants. The incidence varies greatly, depending upon the region of the country and the immigration patterns of that location.

The findings in this report are subject to at least four limitations. First, it was not possible to verify the information that was reported from state NBS programs. Second, complete data were not received from all states, so the findings are only an estimate of the incidence of SCT in the United States. Third, the part of the study that focused on incidence for different races and ethnicities is limited by how accurately the NBS data reflect the actual race/ethnicity of the infants. Finally, the information that the states provided was based on newborn screening results only. These results were not confirmed diagnoses, and so there might be a small number of incorrect results.

This study shows that as many as 1.5% of infants born in the United States have SCT. SCT is benign for most carriers; however, studies have been published suggesting its association in some persons with various conditions, including renal medullary carcinoma, hematuria, renal papillary necrosis, hyposthenuria, splenic infarction, exercise-related deaths, thromboembolic disease, pregnancy-related complications, complicated hyphema, and acute chest syndrome (7). In addition, persons with SCT are at risk for having children with SCD if their partner also has SCT or one of several other abnormal hemoglobin genes, including Hb C and Hb ß-thalassemia. Persons with SCD, in contrast to SCT, are at risk for several serious complications, including hemolytic anemia, bacterial infections, vaso-occlusive pain crisis, stroke, chronic organ damage, and pulmonary hypertension (8). Based on previous studies, there are no standardized methods or protocols for alerting families or health care providers to this information, educating them about the potential health outcomes that might be associated with the condition, or counseling them about the impact that this might have on the family’s future reproductive choices. By including educational materials and providing genetic counseling at the same time that families are provided positive SCT results, the occurrence and public health burden of SCD might be reduced.

## Figures and Tables

**TABLE 1:** Incidence of sickle cell trait (SCT) — 44 U.S. states, 2010

State	No. of infants screened	No. of infants with a positive SCT screen result	Incidence per 1,000 infants screened
Alabama	58,836	1,923	32.7
Alaska	11,269	56	5.0
Arizona	84,257	477	5.7
Arkansas	39,264	563	14.3
California	498,924	4,113	8.2
Colorado	—	—	—
Connecticut	38,809	648	16.7
Delaware	11,893	258	21.7
District of Columbia	—	—	—
Florida	214,948	5,564	25.9
Georgia	—	—	—
Hawaii	18,940	86	4.5
Idaho	22,803	46	2.0
Illinois	176,634	3,056	17.3
Indiana	84,108	987	11.7
Iowa	37,991	203	5.3
Kansas	41,580	374	9.0
Kentucky	57,977	572	9.9
Louisiana	63,005	1,366	21.7
Maine	—	—	—
Maryland	77,806	2,359	30.3
Massachusetts	72,949	1,042	14.3
Michigan	112,986	2,854	25.3
Minnesota	67,550	535	7.9
Mississippi	39,278	1,341	34.1
Missouri	76,308	1,002	13.1
Montana	11,961	10	0.8
Nebraska	26,176	198	7.6
Nevada	35,687	798	22.4
New Hampshire	13,032	42	3.2
New Jersey	102,660	2,040	19.9
New Mexico	26,146	81	3.1
New York	245,280	5,371	21.9
North Carolina	122,324	2,504	20.5
North Dakota	10,383	21	2.0
Ohio	138,952	2,077	14.9
Oklahoma	—	—	—
Oregon	45,606	177	3.9
Pennsylvania	—	—	—
Rhode Island	11,791	182	15.4
South Carolina	55,813	1,650	29.6
South Dakota	12,334	79	6.4
Tennessee	84,533	2,411	28.5
Texas	390,611	4,972	12.7
Utah	51,486	126	2.4
Vermont	5,702	24	4.2
Virginia	97,528	1,865	19.1
Washington	83,086	448	5.4
West Virginia	29,928	81	2.7
Wisconsin	67,163	676	10.1
Wyoming	—	—	—
**Overall (44 states)**	**3,576,297**	**55,258**	**15.5**

**TABLE 2:** Incidence of sickle cell trait (SCT), by Hispanic ethnicity — 13 U.S. states, 2010

State	No. of infants screened	No. of infants with a positive SCT screen result	Incidence per 1,000 infants screened
California	262,238	1,542	5.9
Florida	59,763	582	9.7
Hawaii	252	16	63.5
Idaho	3,696	11	3.0
Kansas	6,479	48	7.4
Louisiana	1,981	19	9.6
Minnesota	4,990	47	9.4
Missouri	3,744	16	4.3
Montana	429	2	4.7
Nevada	12,361	162	13.1
New Hampshire	504	2	4.0
Washington	15,537	115	7.4
West Virginia	239	2	8.4
**Overall (13 states)**	**372,214**	**2,564**	**6.9**

**TABLE 3:** Incidence of sickle cell trait (SCT), by race — 13 U.S. states, 2010

State	Asian, Native Hawaiian, or Other Pacific Islander			Black or African American			White		
		
	No. of infants screened	No. of infants with a positive SCT screen result	Incidence per 1,000 infants screened	No. of infants screened	No. of infants with a positive SCT screen result	Incidence per 1,000 infants screened	No. of infants screened	No. of infants with a positive SCT screen result	Incidence per 1,000 infants screened
Alabama	567	0	0.0	17,616	1,728	98.1	34,670	145	4.2
California	52,018	54	1.0	30,575	2,103	68.8	384,092	1,551	4.0
Kansas	1,206	2	1.7	3,026	221	73.0	33,979	105	3.1
Louisiana	0	—	—	24,307	1,204	49.5	35,632	124	3.5
Michigan	2,384	74	31.0	20,315	2,048	100.8	71,295	263	3.7
Minnesota	4,167	15	3.6	5,356	331	61.8	48,484	71	1.5
Mississippi	274	3	10.9	17,675	1,255	71.0	19,500	64	3.3
Missouri	940	2	2.1	11,059	805	72.8	56,254	79	1.4
Montana	138	0	0.0	74	3	40.5	10,331	5	0.5
New Hampshire	421	1	2.4	182	8	44.0	11,623	27	2.3
Ohio	2,565	10	3.9	21,401	1,541	72.0	100,116	226	2.3
Washington	8,433	2	0.2	4,221	175	41.5	67,391	56	0.8
West Virginia	137	1	7.3	925	39	42.2	26,319	13	0.5
**Total (13 states)**	**73,250**	**164**	**2.2**	**156,732**	**11,461**	**73.1**	**899,686**	**2,729**	**3.0**

## Footnotes

* Categories were Hispanic, non-Hispanic, or not available.

† Categories were American Indian/Alaska Native only; Asian or Native Hawaiian or Other Pacific Islander only; black or African American only; white only; more than one race; and other.

## References

[1] Kavanagh PL, Wang CJ, Therrell BL, Sprinz PG, Bauchner H (2008). Communication of positive newborn screening results for sickle cell disease and sickle cell trait: variation across states. Am J Med Genet C Semin Med Genet.

[2] Christopher SA, Collins JL, Farrell MH (2012). Effort required to contact primary care providers after newborn screening identifies sickle cell trait. J Natl Med Assoc.

[3] Kladny B, Williams A, Gupta A, Gettig EA, Krishnamurti L (2011). Genetic counseling following the detection of hemoglobinopathy trait on the newborn screen is well received, improves knowledge, and relieves anxiety. Genet Med.

[4] Benson JM, Therrell BL (2010). History and current status of newborn screening for hemoglobinopathies. Semin Perinatol.

[5] Lorey FW, Arnopp J, Cunningham GC (1996). Distribution of hemoglobinopathy variants by ethnicity in a multiethnic state. Genet Epidemiol.

[6] Derebail VK, Nachman PH, Key NS, Ansede H, Falk RJ, Kshirsagar AV (2010). High prevalence of sickle cell trait in African Americans with ESRD. J Am Soc Nephrol.

[7] Tsaras G, Owusu-Ansah A, Boateng FO, Amoateng-Adjepong Y (2009). Complications associated with sickle cell trait: a brief narrative review. Am J Med.

[8] Hoppe CC (2011). Newborn screening for hemoglobin disorders. Hemoglobin.

